# Emergence of local synchronization in neuronal networks with adaptive couplings

**DOI:** 10.1371/journal.pone.0178975

**Published:** 2017-06-02

**Authors:** Shilpa Chakravartula, Premananda Indic, Bala Sundaram, Timothy Killingback

**Affiliations:** 1 Center for Vascular Biology Research, Beth Israel Deaconess Medical Center, Harvard Medical School, Boston, MA, United States of America; 2 Department of Electrical Engineering, University of Texas, Tyler, United States of America; 3 Department of Physics, University of Massachusetts, Boston, United States of America; 4 Department of Mathematics, University of Massachusetts, Boston, United States of America; Lanzhou University of Technology, CHINA

## Abstract

Local synchronization, both prolonged and transient, of oscillatory neuronal behavior in cortical networks plays a fundamental role in many aspects of perception and cognition. Here we study networks of Hindmarsh-Rose neurons with a new type of adaptive coupling, and show that these networks naturally produce both permanent and transient synchronization of local clusters of neurons. These deterministic systems exhibit complex dynamics with 1/*f*^*η*^ power spectra, which appears to be a consequence of a novel form of self-organized criticality.

## Introduction

Synchronization is ubiquitous in nature [1]. Examples of this phenomenon are: synchronization in arrays of laser [2, 3] and microwave oscillators [4], and in superconducting Josephson junctions [5–7], the synchronized beating of wings in a flock of birds [8], the chirping of crickets in unison [9], and the synchronized clapping of a crowd [10]. Synchronization is of great importance in neuroscience [11]. There is strong experimental evidence that synchronization of neuronal oscillatory activity is a central mechanism in a variety of cognitive and perceptual processes in the brain, including: the functioning of working memory, the routing of signals across cortical networks, sensory motor awareness, and perceptual grouping [12–14]. Synchronization has been observed experimentally between areas of the visual cortex and parietal cortex in awake cats during a visual-motor integration task [14]. The occurrence of synchronization in a cognitive task in humans has also been demonstrated experimentally [15]. Studies have also found that stored memory patterns depend on spontaneously occurring synchrony in neuronal networks [16]. Moreover, abnormally synchronized neural activity has been implicated in a number of clinical disorders, including: schizophrenia, epileptic seizures, and Parkinson’s disease [17]. Understanding these phenomena is of fundamental importance in neuroscience, and this motivates the present study on synchronization in neuronal networks.

Previous theoretical studies of neural synchronization have largely focused on the issue of global synchronization, i.e., studying the conditions under which all neurons in the network behave in unison [18]. However, it is known from experimental neuroscience that global synchronization does not occur in normal brains. For example, it has been found experimentally that the visual recognition system of the cat and macaque monkey show local synchronization within brain regions [19]. It has also be found that local synchronization, both prolonged and transient, plays a crucial role in many aspects of memory processes [20]. The purpose of this paper is to study how local synchronization occurs in adaptively coupled networks of neurons. The effect on synchronization of variable couplings in networks of simple oscillators has previously been explored in the literature [21–23]. Here we develop this research theme by introducing a new type of adaptive coupling between biologically realistic neurons, and show that it naturally results in the emergence of locally synchronized group of neurons.

## Model

Networks of biologically-inspired neurons provide a natural theoretical framework for studying neural synchronization [24]. Here we model individual neurons using the Hindmarsh-Rose equations [25, 26]:

$$\begin{array}{ccc} \dot{x} & = & y-x^{3}+bx^{2}-z+I, \end{array}$$ (1)

$$\begin{array}{ccc} \dot{y} & = & 1-5x^{2}-y, \end{array}$$ (2)

$$\begin{array}{ccc} \dot{z} & = & r[4\left(x-x_{0}\right)-z]. \end{array}$$ (3)

Eqs (1) and (2) model the fast dynamics associated with a neuron, whereas Eq (3) describes the slow dynamics. The variable *x*(*t*) describes the membrane potential; *y*(*t*), the fast current, models the transport of potassium and sodium ions across the fast ion channel; and *z*(*t*), the slow current, models the transport of other ions through the slow channels. The parameters are as follows: *I* specifies the membrane input current to the neuron; *b* allows for switching between spiking and bursting behavior and also controls the frequency of spiking; *r* controls the rate of change of the slow variable *z*(*t*) and, if spiking is present, it determines the spiking frequency, while if bursting is present, it governs the number of spikes per burst; and *x*_0_ determines the resting potential for the neuron. We fix the parameters *b*, *r* and *x*_0_ to the standard values *b* = 3.0, *r* = 0.006 and *x*_0_ = −1.6 [27]. Furthermore, we set *I* = 2.8, which corresponds to bursting neurons [27, 28], since this behavior seems to be an essential component of information processing in the brain [29, 30].

We consider a network Γ of *N* identical Hindmarsh-Rose neurons coupled through electrical synapses (i.e., via gap junctions, which are known to play a fundamental role in a wide range of neural systems, including the mammalian brain [24, 31, 32], and to be particularly important in maintaining neural synchronization [33–38]). The coupled system of neurons is described by the following equations:

$$\begin{array}{ccc} {\dot{x}}_{i} & = & y_{i}-x_{i}^{3}+bx_{i}^{2}-z_{i}+I+\sum_{j=1}^{N}A_{ij}k_{ij}\left(x_{j}-x_{i}\right), \end{array}$$ (4)

$$\begin{array}{ccc} {\dot{y}}_{i} & = & 1-5x_{i}^{2}-y_{i}, \end{array}$$ (5)

$$\begin{array}{ccc} {\dot{z}}_{i} & = & r[4\left(x_{i}-x_{0}\right)-z_{i}]. \end{array}$$ (6)

Here *x*_*i*_(*t*), *y*_*i*_(*t*) and *z*_*i*_(*t*) denote the *x*(*t*), *y*(*t*) and *z*(*t*) variables of the *i*th neuron, respectively, *k*_*ij*_(*t*) (where *k*_*ij*_(*t*) ≥ 0, for all *i*, *j* and *t*) represents the coupling between neurons *i* and *j* at time *t*, and *A* = (*A*_*ij*_) is the adjacency matrix of the network Γ (i.e., *A*_*ij*_ = 1 if neurons *i* and *j* are connected by an edge in Γ, and *A*_*ij*_ = 0 otherwise). For *k*_*ij*_(*t*) = *k*, for all *i*, *j*, and Γ the complete network, Eqs (4)–(6) describes *N* Hindmarsh-Rose neurons, each of which is coupled to all others with common, fixed, coupling strength *k* [27, 39–41].

In this paper we investigate the effects of connection plasticity on the dynamics of the neuronal network defined by Eqs (4)–(6). Previous approaches to modeling connection plasticity between neurons have focused on properties, such as long-term potentiation (LTP) [42, 43] and spike-timing-dependent plasticity (STDP) [44], which are particular to chemical synapses. Here, in contrast, we consider connection plasticity in networks of neurons coupled through electrical synapses. We allow the strength of the coupling *k*_*ij*_(*t*) between adjacent neurons *i* and *j* to depend on the states (i.e., the membrane potentials) of the neurons. This assumption is natural since it is known that the electrical coupling between neurons via gap junctions may be modulated by neural activity [45–49]. The form of the dependence that we assume is motivated by Hebb’s law that synaptic connections are strengthened between neurons that are active simultaneously [50]. Thus, we assume that the coupling *k*_*ij*_(*t*) between *i* and *j* is adaptive, and will increase in strength if *i* and *j* are in (approximately) the same state and will decrease if *i* and *j* are in dissimilar states. Precisely, we take the time evolution of the coupling *k*_*ij*_(*t*) between adjacent neurons *i* and *j* to be governed by the equation

$$\begin{array}{c} {\dot{k}}_{ij}=k_{ij}\left[\alpha e^{-\beta {\left(x_{i}-x_{j}\right)}^{2}}-\gamma \left(k_{ij}+1\right)\right], \end{array}$$ (7)

where *α*, *β* and *γ* are positive parameters. Thus, with *k*_*ij*_ determined by Eq (7), the last term in Eq (4) defines an adaptive diffusive coupling in the network of Hindmarsh-Rose neurons.

We note some features of the dynamics of Eq (7). (A) If two neurons *i* and *j* are unsynchronized (i.e., *x*_*i*_ is very different from *x*_*j*_) then Eq (7) reduces to ${\dot{k}}_{ij}\approx -\gamma k_{ij}\left(k_{ij}+1\right)<0$, and the strength of the coupling between *i* and *j* decreases, and consequently *i* and *j* remain unsynchronized. (B) Note that, ${\hat{k}}_{ij}=0$ is always an equilibrium of Eq (7). For 0 < *k*_*ij*_ ≪ 1, Eq (7) becomes ${\dot{k}}_{ij}\approx k_{ij}\left(\alpha e^{-\beta {\left(x_{i}-x_{j}\right)}^{2}}-\gamma \right)$. For such values of *k*_*ij*_, if neurons *i* and *j* are synchronized (i.e., *x*_*i*_ = *x*_*j*_) then Eq (7) further reduces to ${\dot{k}}_{ij}\approx k_{ij}\left(\alpha -\gamma \right)$; thus we require that *α* > *γ* to ensure that ${\dot{k}}_{ij}>0$ (and thus ${\hat{k}}_{ij}$ is unstable) for synchronized neurons, ensuring that the coupling between *i* and *j* increases, maintaining their synchronization. (C) We also note that, for synchronized neurons *i* and *j* (i.e., *x*_*i*_ = *x*_*j*_), Eq (7) has a second equilibrium, $k_{ij}^{*}\neq 0$, such that $\alpha -\gamma (k_{ij}^{*}+1)=0$. Thus, $k_{ij}^{*}=\frac{\alpha }{\gamma }-1$. Eqs (4)–(7) model the adaptively coupled neuronal network that we study here. We observe that the form of the variation in coupling strength between neurons that we have postulated has the effect of increasing the synchronization between neurons that are already in similar states and of further decreasing the synchronization between neurons in dissimilar states.

It would be very interesting to consider the full stability analysis of Eqs (4)–(7), however, this appears to be a highly non-trivial enterprise which is beyond the scope of the current work. While it would be most illuminating to understand the fixed points of this system and the possible bifurcation that can occur as the parameters *α*, *β* and *γ* vary, achieving such an understanding would seem to be quite ambitious as even the fixed-point equations constitute a high dimensional system of coupled transcendental equations. As a possibly more manageable first step for future work in this direction it may be worth considering the stability and bifurcation analysis of a single pair of adaptive coupled neurons satisfying Eqs (4)–(7).

## Results

We now present results obtained by numerically integrating this neuronal network. We consider the time evolution of the network for initial coupling strengths *k*_*ij*_ chosen uniformly randomly from (0, 1). Here we fix *α* = 1, *γ* = 0.5 (so *k*_*ij*_ ∈ [0, 1], for all *i*, *j*) and treat *β* as a control parameter. The results described here are for *β* = 12, although we obtain qualitatively similar results for the range of values *β* ∈ [10, 12.5].

**Fig 1:**
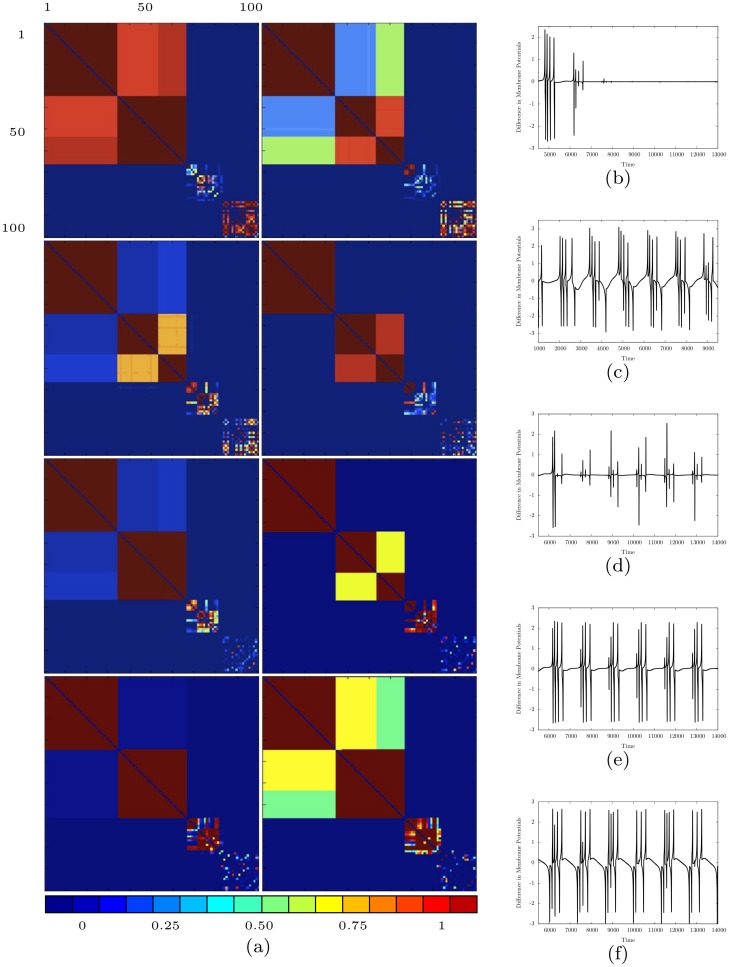
The time-evolution of coupling strengths and membrane potentials for a network of 100 neurons for *I* = 2.8 and *β* = 12. (a) The coupling strength matrix. Starting from a random initial condition (not shown), consecutive pictures from left to right and top to bottom are for times *t* = 3800, 3810, 3820, 3830, 3840, 3850, 3860, and 3870. Blue (left end of colorbar) corresponds to *k*_*ij*_ = 0 and red (right end of colorbar) corresponds to *k*_*ij*_ = 1. The emergence of strong couplings, both permanent and transient, results in the corresponding neurons being permanently or transiently synchronized. The time evolution of the difference between the membrane potentials of pairs of neurons are also shown for: (b) a completely synchronized pair, (c) a completely unsynchronized pair, and (d)—(f) three transiently synchronized pairs.

**Fig 2:**
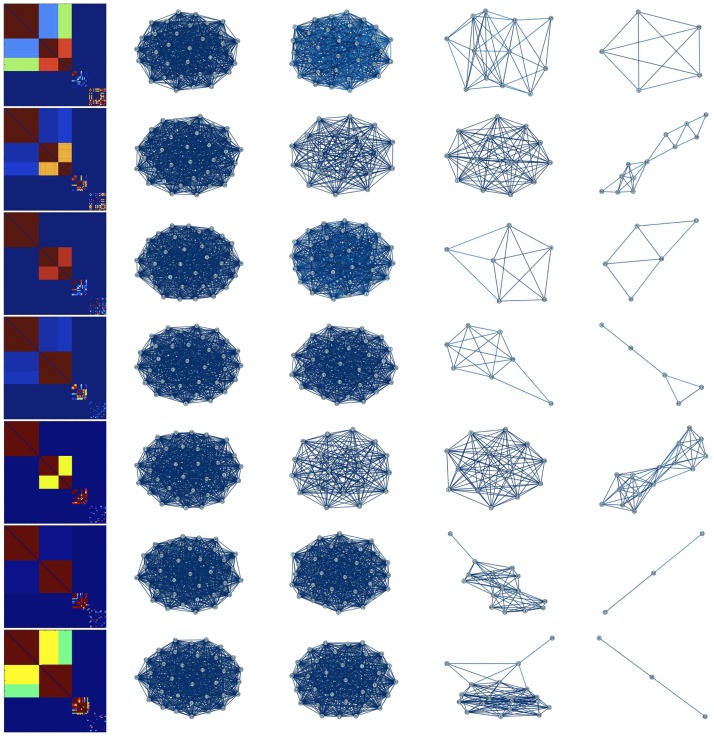
The four largest synchronized groups of neurons at different times for *I* = 2.8 and *β* = 12. In each row, the first picture shows the coupling strength matrix and the four consecutive pictures show the largest synchronized clusters at that time. The first row corresponds to time *t* = 3810, and the time interval between each successive row is 10 units. Permanently and transiently synchronized clusters of neurons are clearly apparent.

**Fig 3:**
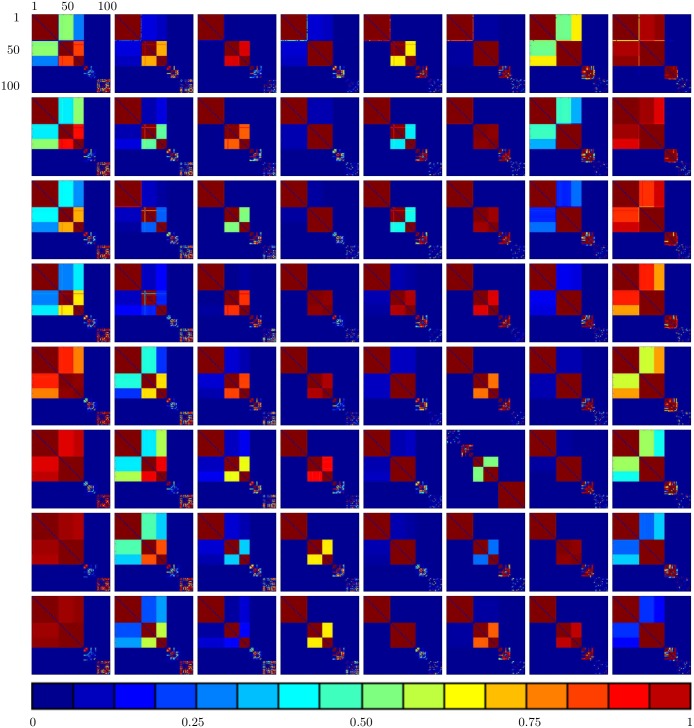
The coupling strength matrix for *I* = 2.8 and *β* = 12 over a longer time interval. The picture at the top right is for time *t* = 3540. The time difference between successive pictures in each row is 10, and the time difference between successive rows is 130. The emergence of both permanent and transient strong couplings, resulting in either permanent or transient synchronization of the corresponding neurons, is apparent throughout the whole time interval studied.

We first consider the case in which Γ is the complete network (i.e., *A*_*ij*_ = 1, for all *i* ≠ *j*). Thus, every neuron is potentially coupled to every other neuron. Fig 1 shows the time evolution of the coupling strengths for a network of *N* = 100 neurons. We observe that over time the coupling *k*_*ij*_(*t*) between any pair of neurons *i*, *j* evolves into one of the following three classes: (1) *k*_*ij*_(*t*) → 1 as *t* → ∞; (2) *k*_*ij*_(*t*) → 0 as *t* → ∞; or (3) *k*_*ij*_(*t*) undergoes sustained oscillations between 0 and 1. This coupling dynamics induces a corresponding synchronization dynamics for the neurons: (1) pairs of neurons *i*, *j* for which *k*_*ij*_(*t*) → 1 as *t* → ∞ become completely synchronized (i.e., |*x*_*i*_(*t*) − *x*_*j*_(*t*)| → 0 as *t* → ∞); (2) *i*, *j* for which *k*_*ij*_(*t*) → 0 as *t* → ∞ become completely unsynchronized; and (3) *i*, *j* for which *k*_*ij*_(*t*) continuously varies between 0 and 1 become synchronized when *k*_*ij*_(*t*) is high (greater than approximately 0.8) and then lose synchronization as *k*_*ij*_(*t*) falls. The variations in the couplings result in the emergence over time of clusters of both permanently and transiently synchronized neurons. Within each cluster of permanently synchronized neurons, the couplings between neurons evolve dynamically to 1. For transiently synchronized neurons, the couplings between such neurons continuously change between 0 and 1 under the dynamics, resulting in the emergence of clusters of neurons that are synchronized for a period of time when the couplings are high and then become desynchronized as the couplings decrease. In addition, the couplings between some neurons evolve dynamically to 0, resulting in these neurons being permanently unsynchronized. Fig 2 shows the structure of the four largest synchronized clusters in the network at different stages in the time evolution. These clusters are obtained by eliminating edges in the original network with coupling strength less than 0.8. The emergence of permanently and transiently synchronized clusters of neurons is clearly apparent. The time evolution of the coupling strengths over a longer time interval is shown in Fig 3, which makes clear the emergence of both permanent and transient strong couplings, which result in the formation of both permanently and transiently synchronized clusters of neurons, throughout the time interval.

**Fig 4:**
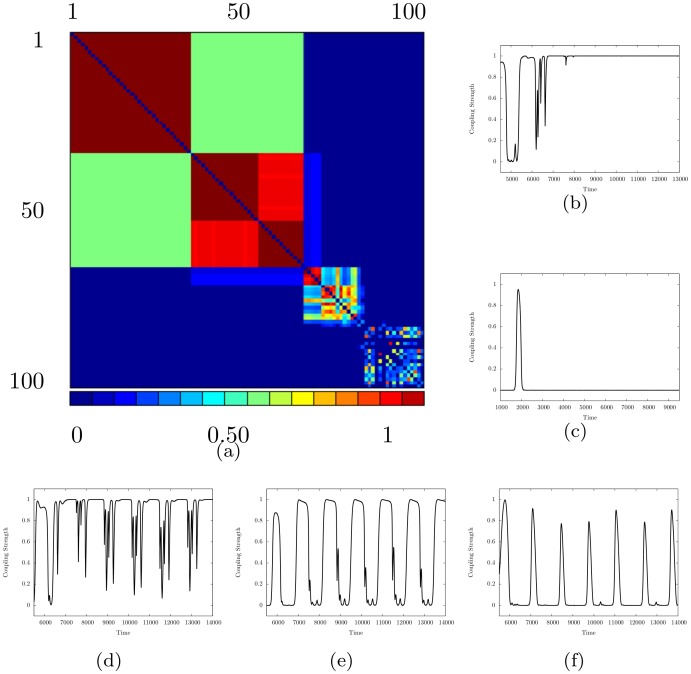
The time-evolution of coupling strengths for *I* = 2.8 and *β* = 12. (a) The time-averaged coupling strength matrix. The average is taken over the time interval [3000, 4000]. Red (right end of colorbar) blocks show couplings between completely synchronized pairs of neurons (〈*k*_*ij*_〉 = 1), blue (left end of colorbar) blocks show completely unsynchronized pairs of neurons (〈*k*_*ij*_〉 = 0), and other colors correspond to couplings between transiently synchronized pairs of neurons (〈*k*_*ij*_〉 ∈ (0, 1)). The time evolution of the coupling strength between pairs of neurons are also shown for: (b) a completely synchronized pair, (c) a completely unsynchronized pair, (d)—(f) three transiently synchronized pairs. The results shown in (b)—(f) are for the same pairs of neurons as the results shown in the corresponding panels of Fig 1.

The nature of the local synchronization process may be elucidated by considering the long-term dynamics of the couplings between neurons in the network. We define the time-average coupling 〈*k*_*ij*_〉 between neurons *i* and *j* over the interval [*T*_0_, *T*] to be $\langle k_{ij}\rangle =\frac{1}{T-T_{0}}\sum_{t=T_{0}}^{T}A_{ij}k_{ij}\left(t\right)$. The time-averages of the couplings between all neurons are shown in Fig 4. This figure clearly illustrates the trichotomy that all pairs of neurons (*i*, *j*) are either: (1) completely synchronized (〈*k*_*ij*_〉 = 1); (2) completely unsynchronized (〈*k*_*ij*_〉 = 0); or (3) transiently synchronized (〈*k*_*ij*_〉 ∈ (0, 1)). The time evolution of *k*_*ij*_(*t*) for pairs of neurons in each of these classes is also shown in Fig 4. The couplings between completely synchronized and completely unsynchronized pairs of neurons reach a steady state, while those between transiently synchronized pairs of neurons continue to evolve in time and drive a complex dynamical system.

**Fig 5:**
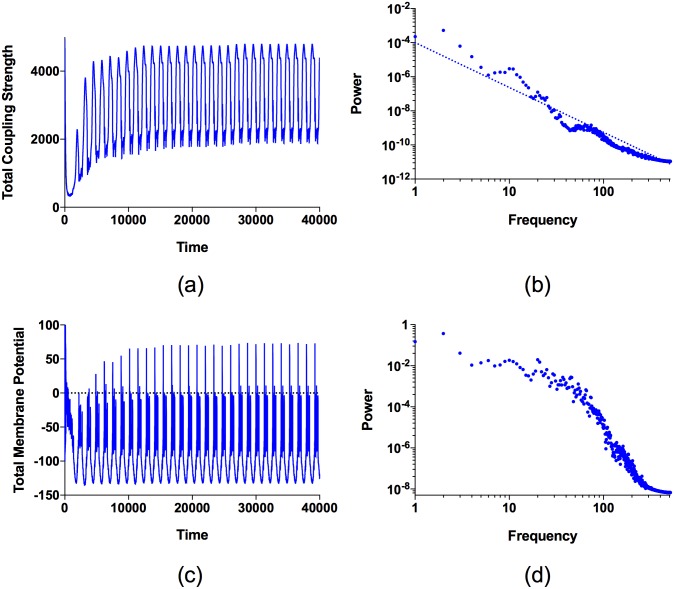
The time series and the Fourier power spectrum of the total coupling strength *K*(*t*) and the total membrane potential *X*(*t*) for *I* = 2.8 and *β* = 12. (a) The time series of *K*(*t*) showns continual variations between higher and lower total coupling strengths. (b) For *K*(*t*), the power *P*(*f*) associated with frequency *f* satisfies to a good approximation the power-law *P*(*f*) ∝ 1/*f*^*η*^, where *η* = 2.628 ± 0.002, indicating that variations in *K*(*t*) occur on all time scales. The dashed line indicates the power-law relation. The large amplitude variation in the time series of *K*(*t*) result in a peak in the power spectrum at *f* ≈ 12, while the smaller amplitude, higher frequency, variations at the bottom of each large amplitude cycle give a secondary peak at frequency *f* ≈ 70. (c) The time series of *X*(*t*) also shows continual variations. (d) For *X*(*t*), the deviations of the power spectrum from a power-law are greater than for *K*(*t*), however, *X*(*t*) also shows variations on a wide range of time scales. The large amplitude oscillation in *X*(*t*) results in a peak in the power spectrum at *f* ≈ 20.

The statistical features of the time evolution of the couplings between neurons can be analyzed by studying the power spectrum of the sum of the connection strengths $K\left(t\right)=\sum_{i,j=1}^{N}A_{ij}k_{ij}\left(t\right)$, which conveniently includes the time-dependence of all couplings between neurons in the network. The time series and power spectrum of *K*(*t*) are shown in Fig 5(a) and 5(b), respectively. The perpetual variation in the total coupling strength *K*(*t*), apparent in Fig 5(a), reflects the dynamic in which groups of neurons transiently synchronize when the couplings between them are strong, subsequently desynchronize as the coupling strengths fall, and later resynchronize as the coupling strengths rise again. We note, from Fig 5(b), that the power *P*(*f*) associated with frequency *f* satisfies, to a good approximation, the power-law *P*(*f*) ∝ 1/*f*^*η*^, where *η* = 2.628 ± 0.002. The fact that the power spectrum satisfies a 1/*f*^*η*^ power law implies that variations in *K*(*t*) occur on all time scales. We find the same 1/*f*^*η*^ power-law for all values of *β* ∈ [10, 12.5], the range of *β*-values for which local synchronization occurs.

We have also investigated the time series and power spectrum of the total membrane potential $X\left(t\right)=\sum_{i=1}^{N}x_{i}\left(t\right)$, which is shown in Fig 5(c) and 5(d), respectively. The power spectrum of *X*(*t*) has greater deviations from a power law than that of *K*(*t*), however, *X*(*t*) also exhibits variations on a wide range of time scales.

**Fig 6:**
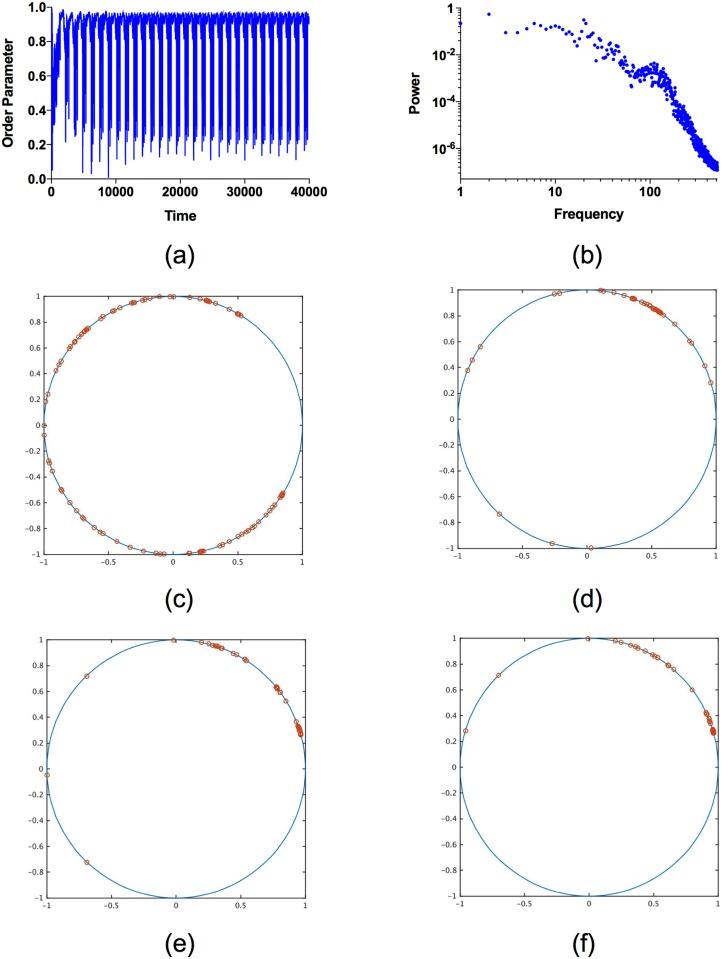
The time variation in the order parameter *χ*(*t*) for *I* = 2.8 and *β* = 12. (a) The time series of *χ*(*t*) shows sustained variations between values close to 1, corresponding to high levels of synchronization, and values close to 0, corresponding to lower levels of synchronization. The continual variation of *χ*(*t*) results from the repeated transient synchronization of neurons in the network. (b) The power spectrum of *χ*(*t*) shows variations on a wide range of time scales, indicative of the complex dynamics apparent in the time series of *χ*(*t*). The large amplitude oscillation in *χ*(*t*) results in a peak in the power spectrum at *f* ≈ 20, while smaller amplitude, higher frequency, variations give a secondary peak at *f* ≈ 100. (c)-(f)The distributions of the complex numbers that define the order parameter on the unit circle in the complex plane at different times. At time *t* = 13 (shown in (c)) the system is largely unsynchronized, and subsequently evolves in time to successively more synchronized states at *t* = 3825 (shown in (d)), *t* = 3910 (shown in (e)), and *t* = 4000 (shown in (f)).

The dynamics of the emergence of permanently and transiently synchronized groups of neurons in the adaptively coupled network is considerably elucidated by considering an appropriate order parameter. Here we define the order parameter $\chi \left(t\right)=\frac{1}{N}\left|\sum_{j=1}^{N}e^{2\pi i{\hat{x}}_{j}\left(t\right)}\right|$, where ${\hat{x}}_{j}\left(t\right)=\frac{x_{j}\left(t\right)+x_{m}}{x_{M}+x_{m}}$ is the rescaled membrane potential (defined using the maximum and minimum values of the membrain potential over the time interval of interest, *x*_*M*_ and *x*_*m*_, respectively), which satisfies ${\hat{x}}_{j}\left(t\right)\in \left[0,1\right]$. The order parameter *χ*(*t*) ∈ [0, 1], for all *t*. Complete global synchronization of all neurons corresponds to *χ*(*t*) = 1, while total lack of synchronization among all neurons results in *χ*(*t*) ≈ 0. Different aspects of the time variation of the order parameter are shown in Fig 6. The time series of *χ*(*t*) is shown in Fig 6(a). The continual variation in *χ*(*t*), apparent in Fig 6(a), vividly illustrates the dynamic of repeated synchronization and desynchronization of clusters of transiently synchronized neurons in the adaptively coupled network. The power spectrum of *χ*(*t*), shown in Fig 6(b), shows greater deviations from a power law than that for *K*(*t*), but still exhibits variations on a large range of time scales, indicative of the complex dynamics of *χ*(*t*). The distributions of the complex numbers ${\left\{e^{2\pi i{\hat{x}}_{j}\left(t\right)}\right\}}_{j=1}^{N}$ on the unit circle in the complex plane at different times are shown in Fig 6(c)–6(f). The configuration shown in Fig 6(c) corresponds to a state of the neuronal network with low levels of synchronization, which then evolves in time to successively more synchronized states, shown in Fig 6(d)–6(f). The system subsequently evolves in time to less synchronized states, followed by more synchronized states, and perpetually repeats this pattern of variation.

We have also studied the coupled neuronal network introduced here for a large variety of model and empirical network topologies Γ. Here we shall only briefly describe certain of these results deferring a more detailed account of these cases to a subsequent publication.

We find that the coupled neuronal network defined on a wide diversity of network topologies Γ, including small-world [51] and scale-free [52] topologies, exhibit exactly analogous behavior to that found above for the complete network topology. We again see that the time evolution of the coupling *k*_*ij*_(*t*) between every pair of adjacent neurons *i* and *j* in Γ falls into one of three classes: (1) *k*_*ij*_(*t*) → 1 as *t* → ∞, resulting in the neurons *i* and *j* becoming completely synchronized; (2) *k*_*ij*_(*t*) → 0 as *t* → ∞, results in *i* and *j* becoming completely unsynchronized; or (3) *k*_*ij*_(*t*) maintains continued oscillations between 0 and 1, results in *i* and *j* being transiently synchronized. The variations in the coupling between adjacent neurons results in the formation over time of clusters of both the permanently and transiently synchronized neurons in Γ.

**Fig 7:**
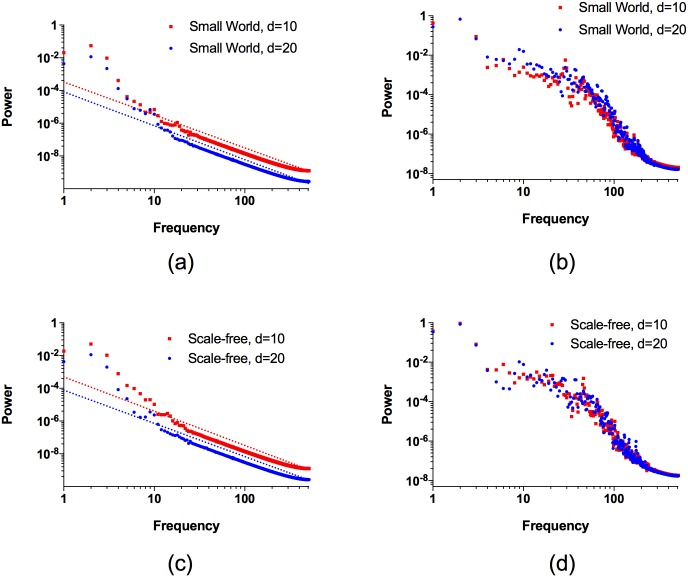
The Fourier power spectrum of the total coupling strength *K*(*t*) and the total membrane potential *X*(*t*) for small-world and scale-free networks for *I* = 2.8 and *β* = 12. (a) For *K*(*t*) on small-world networks with rewiring probability 0.3 the power *P*(*f*) associated with frequency *f* satisfies a power-law *P*(*f*) ∝ 1/*f*^*η*^, where *η* = 2.013 ± 0.002 and *η* = 2.070 ± 0.002 for both mean degree 10 (red squares) and 20 (blue circles), respectively. (b) For *X*(*t*) on the same small-world networks as in (a) the deviations of the power spectrum from a power-law are greater than for *K*(*t*), however, *X*(*t*) also shows variations on a wide range of time scales. (c) For *K*(*t*) on scale-free networks *P*(*f*) satisfies a power-law *P*(*f*) ∝ 1/*f*^*η*^, where *η* = 2.094 ± 0.002 and *η* = 2.028 ± 0.002 for both mean degree 10 (red squares) and 20 (blue circles), respectively. (d) For *X*(*t*) on the same scale-free networks as in (c) the deviations of the power spectrum from a power-law are greater than for *K*(*t*), however, *X*(*t*) also shows variations over a large range of time scales.

The transiently synchronized neurons result in a complex dynamics in which the power spectrum of the total coupling *K*(*t*) satisfies to an excellent approximation a power-law *P*(*f*) ∝ 1/*f*^*η*^. The power spectra for networks Γ of *N* = 100 neurons with small-world and scale-free topologies are shown in Fig 7. For small-world topologies with rewiring probability 0.3 and mean degree 10 and 20, the power spectra, shown in Fig 7(a), satisfy the power-laws *P*(*f*) ∝ 1/*f*^*η*^, with *η* = 2.013 ± 0.002 and *η* = 2.070 ± 0.002, respectively. The power spectra for scale-free topologies with mean degree 10 and 20, shown in Fig 7(c), satisfy the power-laws *P*(*f*) ∝ 1/*f*^*η*^, with *η* = 2.094 ± 0.002 and *η* = 2.028 ± 0.002, respectively. For both small-world and scale-free topologies the power spectra of the total membrane potential *X*(*t*), shown in Fig 7(b) and 7(d), display greater deviations from power-law behavior than *K*(*t*), but nevertheless exhibit variations on a wide range of time scales. It is an intriguing result, worthy of further study, that the power spectrum of *K*(*t*) conforms to a power-law much more closely on complex networks than on the complete network. It is also potentially significant that the power-law exponents are almost equal for quite different complex network topologies, suggesting a possible universality underlying the dynamics of these coupled complex networks of neurons.

## Discussion

Most theoretical studies of synchronization in neuronal networks have focused on understanding the conditions under which global synchronization of neurons occur [18]. However, it is clear empirically that many important aspects of neural behavior depend on local, and often transient, synchronization of groups of neurons in the brain [19]. Here we have studied adaptively coupled neuronal networks, in which the coupling between two neurons is determined dynamically by the states of the neurons, and which has the key feature that the coupling between pairs of adjacent neurons that are in a similar state is strengthened, while that between pairs of adjacent neurons in dissimilar states is weakened. The dynamics of these networks result in the spontaneous emergence of locally synchronized groups of neurons, some of which are permanently synchronized while other show transient synchronization. This pattern of synchronization is found for a significant parameter range in the model. The existence of transient local synchronization (in addition to permanent local synchronization) results in the neuronal couplings having a complex dynamics, which is characterized by the power spectrum *P*(*f*) of the sum of the couplings obeying a power-law *P*(*f*) ∝ 1/*f*^*η*^. For the complete network topology, we find the same power law for all values of the parameter *β* that yields local synchronization. We have also studied the model for other values of *α* and *γ*, and find that for a wide variety of these parameters there exists a significant range of *β* values for which local synchronization occurs and for which the total coupling exhibits a power law spectrum. The exponent of the power law varies with *α* and *γ*, but for fixed *α* and *γ* is independent of *β*. For complex network topologies, including small-world and scale-free topologies, we find no significant variation in the power-law exponents for a wide range of the parameters *α*, *β* and *γ*, which suggests an intriguing universality underlying the dynamics of these adaptively coupled neuronal networks. The 1/*f*^*η*^ power-law in this deterministic model does not depend on any fine tuning of parameters and appears to be a consequence of an interesting new type of self-organized criticality.

The mechanism responsible for driving continuous oscillations in the couplings between pairs of transiently synchronized neurons appears to be a type of dynamical frustration. In this frustration process distinct groups of completely synchronized neurons form in the network, and since the oscillations between different synchronized groups are typically out of phase this results in pairs of neurons spanning different groups having a constantly changing difference in their membrane potentials, which in turn leads to a continuously changing coupling strength between such pairs of neurons. The operation of this process between all pairs of neurons spanning different completely synchronized groups results in many pairs of transiently synchronized neurons with couplings varying on a wide range of time scales. Thus, this deterministic dynamical system apparently self-organizes into a state in which variations in the couplings between transiently synchronized neurons occur on all time scales and, therefore, the emergence of a power-law spectrum in this deterministic model, which does not depend on any fine tuning of parameters, seems to be a consequence of a novel form of self-organized criticality [53]. Concepts of self-organized criticalty, while having been applied to a variety of problems in neuroscience [54–57], including certain aspects of synchronization [58], appears not to have been previously connected to synchronization in networks of neurons adaptively coupled through electric synapses.

The frustration process that we have suggested here to underlie the emergence of a power-law spectrum in an adaptively coupled network of Hindmarsh-Rose neurons is novel in that it depends upon frustration occurring between dynamically formed clusters of neurons, where the neurons within a given cluster are completely synchronized, but neurons lying in different clusters are unsynchronized. We note here that a different, and more conventional type of geometric frustration process plays an important role in other types of neuronal networks [59–62]. Obtaining a deeper understanding of the dynamical frustration process we have suggested for adaptively coupled neuronal networks, and elucidating the connection to power-law spectra seems to be an important task for future research.

In this paper we have considered adaptively coupled networks of Hindmarsh-Rose neurons, where the membrane input current *I* is set to produce bursting behavior for the neurons, as this is known to be a key aspect of information processing in the brain [30, 63]. We have also explored the behavior of our adaptively coupled networks when the membrane input current *I* is set to give spiking behavior for the neurons. We find that the outcome for spiking neurons is very similar to the case of bursting neurons, with the spontaneous emergence of groups of completely synchronized neurons and also of clusters of transiently synchronized neurons, the latter of which result in a complex dynamical process analogous to that found for bursting neurons.

We should also like to mention that the type of adaptively coupled neuronal networks that we have considered here may be relevant to the problem of pattern selection. It is known that complex pattern formation, including spiral waves, can occur in some neuronal networks and it would be an interesting topic for future research to investigate whether such pattern formation occurs in the adaptively coupled Hindmarsh-Rose networks that we have considered here [64–67].

Finally, we remark that while we have focused here on neuronal synchronization it is possible that the type of adaptive coupling we have postulated may have broader applications to the study of local synchronization in other networked systems.
